# *In Vitro* and *In Vivo* Evaluations of Nano-Hydroxyapatite/Polyamide 66/Glass Fibre (n-HA/PA66/GF) as a Novel Bioactive Bone Screw

**DOI:** 10.1371/journal.pone.0068342

**Published:** 2013-07-08

**Authors:** Bao Su, Xiaohua Peng, Dianming Jiang, Jun Wu, Bo Qiao, Weichao Li, Xiaotong Qi

**Affiliations:** 1 Department of Orthopaedics, The First Affiliated Hospital of Chongqing Medical University, Chongqing, China; 2 Department of Respiratory Medicine, Xinhua Hospital, Shanghai Jiaotong University, School of Medicine, Shanghai, China; University of Illinois at Chicago, United States of America

## Abstract

In this study, we prepared nano-hydroxyapatite/polyamide 66/glass fibre (n-HA/PA66/GF) bioactive bone screws. The microstructure, morphology and coating of the screws were characterised, and the adhesion, proliferation and viability of MC3T3-E1 cells on n-HA/PA66/GF scaffolds were determined using scanning electron microscope, CCK-8 assays and cellular immunofluorescence analysis. The results confirmed that n-HA/PA66/GF scaffolds were biocompatible and had no negative effect on MC3T3-E1 cells in vitro. To investigate the in vivo biocompatibility, internal fixation properties and osteogenesis of the bioactive screws, both n-HA/PA66/GF screws and metallic screws were used to repair intercondylar femur fractures in dogs. General photography, CT examination, micro-CT examination, histological staining and biomechanical assays were performed at 4, 8, 12 and 24 weeks after operation. The n-HA/PA66/GF screws exhibited good biocompatibility, high mechanical strength and extensive osteogenesis in the host bone. Moreover, 24 weeks after implantation, the maximum push-out load of the bioactive screws was greater than that of the metallic screws. As shown by their good cytocompatibility, excellent biomechanical strength and fast formation and ingrowth of new bone, n-HA/PA66/GF screws are thus suitable for orthopaedic clinical applications.

## Introduction

Internal fixation with rigid metallic screws, pins and plates is routine for fracture repair [1]. Metallic fixation provides good fracture reduction and allows patients to exercise the limb soon after surgery [2]. However, in the majority of cases, a second surgical event is needed to remove the metallic implants [3], and a significant number of refractures have been observed after removal [4]. Additionally, the extremely high elastic modulus of metallic implants may lead to a “stress shielding” effect that can be detrimental to the healing process and cause localised osteopenia under and near the plate[5]–[8].

To reduce such complications, biodegradable materials have been used as implants for their biodegradability and biocompatibility in the bone environment [9], [10]. Biodegradable implants provide stable initial fixation and then degrade gradually as they are replaced by newly formed bone. However, the accumulation of localised acidic degradation products may lead to a low local pH and chronic aseptic inflammation [11].

In recent years, magnesium alloys have attracted a great deal of interest as next-generation biodegradable materials [12]. Biodegradable magnesium and its alloys have sufficient strength and elastic modulus values that are close to those of bone. However, the rapid corrosion rate of Mg alloys, the potential toxicity of their alloying elements and the release of hydrogen gas during degradation limit their clinical application [13]–[15].

To our knowledge, previous studies have focused on absorbable or degradable biomaterials, but they have disadvantages discussed above. Therefore, we take another new approach and tried to develop a new non-degradable implant which possesses the following features: bioactivity, ability to heal with host bone tissue and satisfy the fracture fixation requirement, inability to be absorbed and degraded, appropriate biomechanical strength and no requirement for a second surgery. n-HA/PA66 is a novel non-degradable nanometre-scale bioactive composite that we investigated in a previous study for bone repair and reconstruction [16]. These assessments showed that n-HA/PA66 has good biocompatibility and osteoconductivity [17]–[20]. Therefore, we planned to use the n-HA/PA66 as the basis materials to make bioactive screws. Because the mechanical strength of n-HA/PA66 was not sufficient for fixing fractures firmly, we used GF to reinforce the composite and applied an n-HA coating on the moulded screws to maintain their excellent bioactive and mechanical properties. In this study, we collaborated with Sichuan University to develop new, non-degradable nano-hydroxyapatite/polyamide 66/glass fibre (n-HA/PA66/GF) bioactive screws and used them to fix intercondylar femur fractures in dogs. The purpose of this study was to assess the biocompatibility, osteogenesis and fixation properties of the constructs in vitro and in vivo.

## Materials and Methods

### 1. Ethics Statement

The use of animals and the experimental protocols was approved by the Animal Care Committee of The First Affiliated Hospital of Chongqing Medical University (Application No 201213).

### 2. Materials Fabrication

#### 2.1. Preparation of GF reinforced n-HA/PA66 screws

All the chemical reagents used in this work were in analytical reagent level. The n-HA/PA66 composite powder was prepared using the co-precipitation method in ethanol [16], [21]. Then, GF and n-HA/PA66 composite powders were dried at 80°C for 24 h and extruded in a corotating twin-screw extruder (Model TSSJ, Chengdu Keqiang, China). The weight ratio of HA, GF and PA is 2∶3: 5. A temperature ranging from 240°C to 290°C and a screw speed of 200 rpm were used to mix GF with n-HA/PA66 composite sufficiently. The extrudates were cooled in deionised water and pelletized. Then, the pelletized extrudates were dried at 80°C for 24 h in vacuum chamber and molded into 3.5 mm diameter screw specimens by an injection moulding machine (KTC-200, Kinki, China) at a temperature ranged from 250°C to 280°C under 30 MPa pressure. Then, the as-prepared GF reinforced composite screws were cleaned ultrasonically in deionised water and dried at 60°C for 6 h.

#### 2.2. Simulated body fluid (SBF) preparation and soaking

SBF was prepared according to the methods of Kokubo et al [22] and buffered at pH 7.4 with 50 mmol (CH_2_OH)_3_CNH_2_ and 45 mmol HCl. The concentration of prepared SBF is given in Table 1. The GF reinforced screws were immersed in 50 ml SBF for 24 h at 37°C and taken out of the fluid. Finally, the specimens were gently washed with deionised water and dried at room temperature. They were sterilised at a high temperature and pressure before implantation.

**Table 1:** Ion concentration of SBF and human body plasm.

	Ion concentration (mmol/L )							
	Na^+^	K^+^	Ca^2+^	Mg^2+^	Cl^–^	HCO_3_^–^	HPO_4_^2−^	SO_4_^2−^
Human body plasm	142.0	5.0	2.5	1.5	103.0	4.2	1.0	0.5
SBF employed	142.0	5.0	2.75	1.5	148.3	4.2	1.1	0.5

### 3. Scanning Electron Microscope (SEM) Observation

The coating surface and cross sections of the screws were evaluated using a SEM (JSM-7500F, Japan). Prior to examination, each sample was coated with gold. Energy-dispersive X-ray spectroscopy (EDS, 51-XMX, UK) in the SEM was used to analyse the elements of the coating layer.

### 4. In vitro Evaluation

#### 4.1. Cell culture

Pre-osteoblastic MC3T3-E1 cells were obtained from the Chinese Academy of Sciences Cell Bank.Cells were cultured in α-MEM medium (Hyclone, USA) containing 10% FBS (Gibco, USA). Cells were subcultured every 5 days and maintained at 37°C in a humidified incubator with 5% CO_2_.

#### 4.2. Cell seeding and cytocompatibility

Samples of the n-HA/PA66/GF scaffolds were cut into discs of Φ10×1 mm and pre-wetted with 1 ml of FBS-free α-MEM in a 24-well plate overnight. Then the medium was removed, and the MC3T3-E1 cells were seeded on the n-HA/PA66/GF disc at a density of 5.0×10^4^ cells and incubated for 3 or 7 days. To observe the growth and morphology of MC3T3-E1 cells on the surface of each scaffold, cell/scaffold constructs were rinsed with PBS, fixed with 2.5% (v/v) glutaraldehyde and observed with a SEM.

#### 4.3. Cell viability assays

α-MEM supplemented with 10% FBS was used as the extraction medium. The material surface area/extracting vehicle ratio was 3 cm^2^/ml, as specified by ISO 10993–12∶2007. The extracts of PA66 scaffolds and a 64 g/L phenol solution were used as negative and positive controls, respectively. A Cell Count Kit-8 (CCK-8, Beyotime, Jiangsu, China) was employed to quantitatively evaluate cell viability [23]. Briefly, 1.0×10^4^ MC3T3-E1 cells were seeded in 24-well cell culture plates. After cell adhesion was verified, the culture medium was replaced by one of the two types of extracts or phenol solution (1 ml per well). The cells were incubated in the culture plates for 1, 3, 5, 7 or 14 days, and cell morphology was observed using an inverted microscope. Then, the CCK-8 solution was added (20 µl per well) and incubated for 2 h at 37°C and 5% CO_2_. 100 µl of the reacted reagent from each well was transferred to 96-well plates, and the absorbance at 450 nm was determined using a microplate spectrophotometer (MD, SpectraMax M2, USA). Six parallel replicates of each sample at each time point were prepared so that statistics could be performed.

#### 4.4. Cellular immunofluorescence

MC3T3-E1 cell proliferation and distribution on n-HA/PA66/GF scaffolds were studied using confocal microscopy. PA66 scaffolds were used as a negative control. A total of 10^4^ cells were cultured on each scaffold disk for 4 days. Then, the cells/scaffolds were washed with PBS, fixed in 4% paraformaldehyde and washed again with PBS. They were then immersed in 0.1% Triton X-100 and washed in PBS. The cells were blocked with normal goat serum and incubated with anti-F-actin primary antibody (1∶100, Bioss, Peking, China) at 4°C overnight followed by FITC-conjugated secondary antibody (1∶100, Bioss, Peking, China) for 2 h at room temperature. The cells were rinsed with PBS, and the nuclei were stained with propidium iodide (PI). Imaging experiments were conducted using a laser scanning confocal microscope (Leica, DMIRE2, Germany).

### 5. In vivo Evaluation

#### 5.1. Animal model and surgery

Twenty-four healthy, mature dogs were randomly and evenly divided into two groups: n-HA/PA66/GF screws were used in one group, and Φ3.5 mm metallic screws (Tianjin Zhengtian Group Medical Polymer Co., Ltd., China) were used in the other. Each animal served as its own control. General anaesthesia was achieved via intraperitoneal injection (1.0 ml/kg) of 3% pentobarbital.

A longitudinal lateral incision was made, and the knee was exposed layer by layer. The patella was dislocated to expose the condylus lateralis femoris. Then, the condylus lateralis femoris was cut away from the femur to create an intercondylar fracture model of the femur. Either bioactive screws or metallic screws were used to fix the fracture after reduction. Identical drills and tapers were used for all screws. Each of the layers was aligned and sutured after copious physiological saline irrigation. The animals tested were kept in individual cages in standard conditions and allowed to be fully active after surgery. They were euthanised by an intravenous injection of overdose of sodium pentobarbital at 4, 8, 12 or 24 weeks after implantation. The distal femurs were excised immediately after sacrifice.

### 5.2. General Photography and Imaging Examination

General photography and three-dimensional (3D) CT reconstruction of both knees were conducted before the dogs were euthanised at 4, 8, 12 or 24 weeks to observe the fracture healing and the internal fixation performance of the screws.

### 5.3. Micro-computed Tomography (Micro-CT) Examination

Bone mineral density examination of both knees was conducted at 4, 8, 12 or 24 weeks using a Viva CT 40 (Scanco Medical, Switzerland) at 30 µm intervals, with a voltage of 70 kV and 114 µA. Then, the images were imported into the software and reconstructed in 3D format.

### 5.4. Histological Analysis

The distal femur and surrounding tissue were excised, fixed in paraformaldehyde and embedded in methyl methacrylate. Tissue blocks were ground to a thickness of 100 µm with a microtome (Leica, 2600SP, Germany) and stained with toluidine blue (TB). The stained sections were observed using a light microscope (Olympus, BX51, Japan).

### 5.5. Biomechanical Test

The muscles, ligaments, soft tissue and periosteum of the distal femoral specimens from 4, 8, 12 and 24 weeks were completely removed. Five parallel specimens in each group were tested at each time point. The specimens were mechanically tested by three-point bending using a universal mechanical testing system (AG-IC 50 KN, Shimadzu, Japan) at a loading speed of 1 mm min^−1^, and the maximum load of each specimen was recorded.

### 6. Statistical Analysis

Quantitative data were expressed as the mean±SD. Statistical analysis was performed using SPSS 17.0 software. Two experimental groups were evaluated with Student’s t-test. A *p* value <0.05 was considered statistically significant.

## Results

### 1. SEM Observation and EDS Analysis

**Figure 1:**
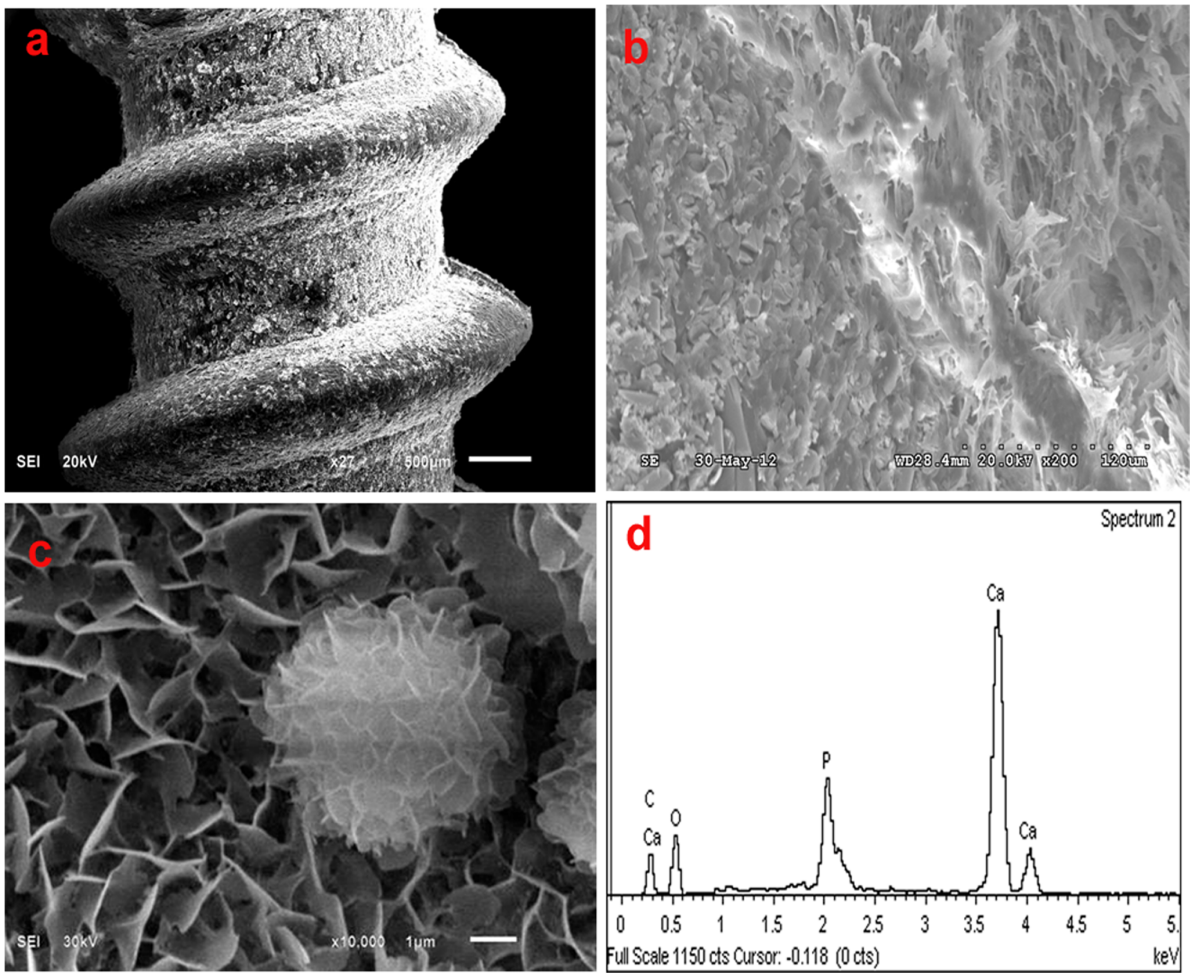
SEM and EDS micrographs of the n-HA/PA66/GF screw. (a) Coating layer. (b) Cross section. (c) n-HA particles on the screw surface. (d) EDS graph of the coating layer.

As shown in Fig. 1a and 1b, there was no apparent interval or phase separation between the bulk matrix and n-HA coating layer, and the coating is distributed evenly around the screw. The coating particles are nanosized (Fig. 1c). The EDS graph (Fig. 1d) shows that the main elements in the coating layer are Ca and P, indicating that the coating layer is n-HA.

### 2. In vitro Evaluations

#### 2.1. Cytocompatibility test

**Figure 2:**
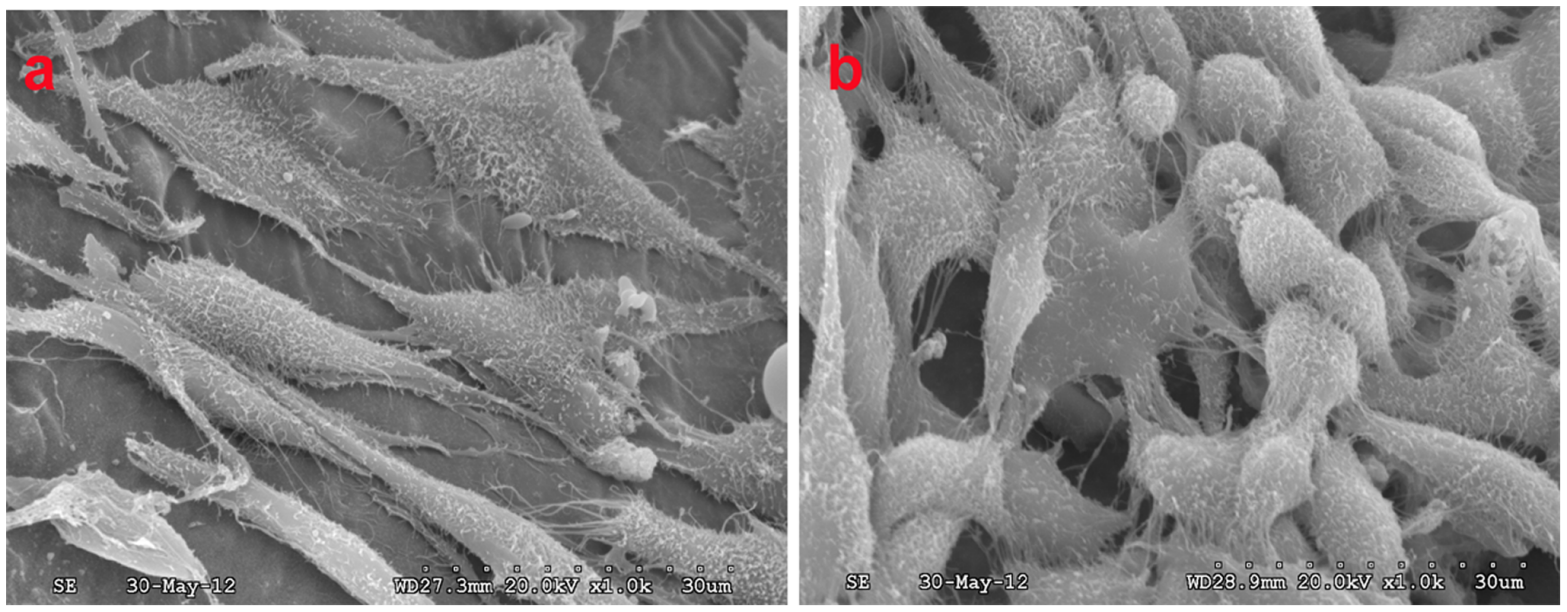
SEM micrographs of the MC3T3-E1 cells cultured on the n-HA/PA/GF scaffold. (a) Long, spindle-shaped cells attached to the walls of the scaffold after 3 days (SEM×1000). (b) A confluent layer of cells proliferated on the scaffold after 7 days (SEM×1000).

Three days after culturing, cells with predominantly long spindle shapes connected with each other and became anchored to the surface of the scaffold via their pseudopodia (Fig. 2a). Seven days after culturing, cells had proliferated dramatically and formed stratified cell layers, accompanied by filamentous fibres on the surface of the scaffold (Fig. 2b).

#### 2.2. Cell viability test

**Figure 3:**
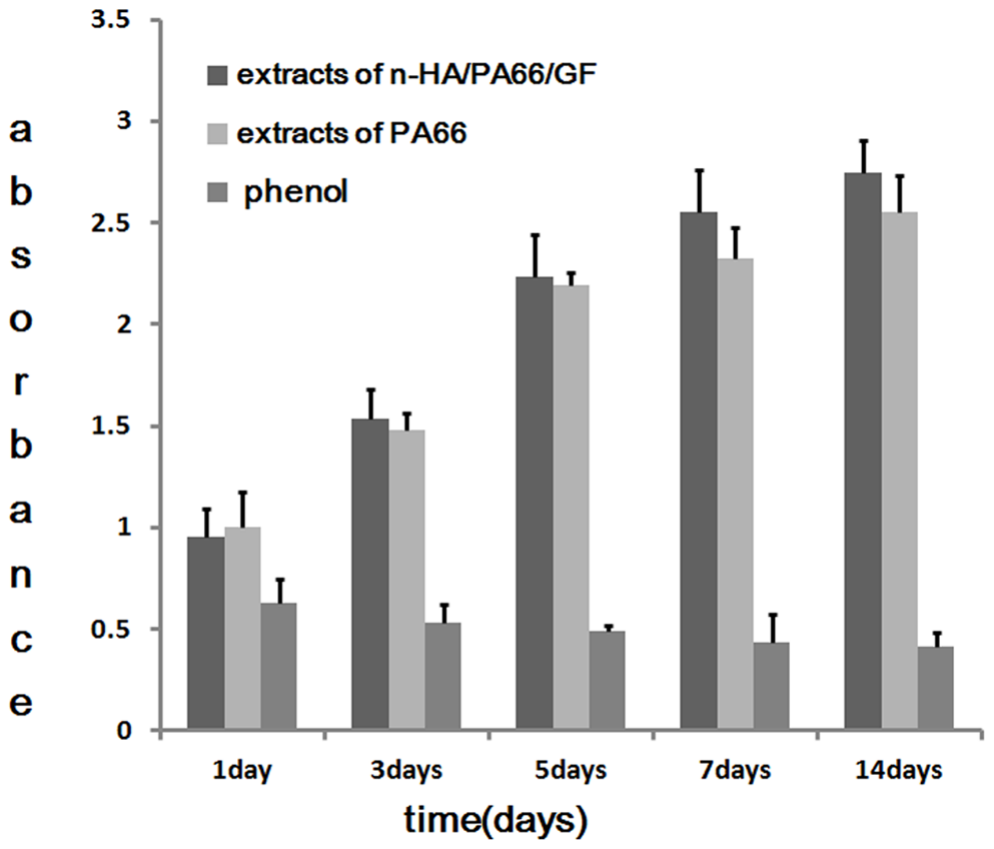
CCK-8 assay for proliferation of MC3T3-E cells cultured with extracts of n-HA/PA66/GF, PA66 (negative control group) and phenol solution (positive controls group) for 1, 3, 5 7 and 14 days.

As shown in Fig. 3, the cell number increased with culture time in each group. Statistical analyses indicated that the cells cultured with the n-HA/PA66/GF sample exhibited the greatest proliferation among the three groups, showing a significant difference from the negative and positive control groups after 5 days of co-culture (*p*<0.05). However, in the first 5 days, there were no statistical differences in cell numbers between the experimental and negative control groups. Cell numbers in the experimental and negative control groups increased dramatically, but the rate of cell proliferation decreased thereafter. Cell proliferation at day 7 and 14 was significantly increased in the experimental group compared with the negative and positive control groups (*p*<0.05).

#### 2.3. Cellular immunofluorescence assays

**Figure 4:**
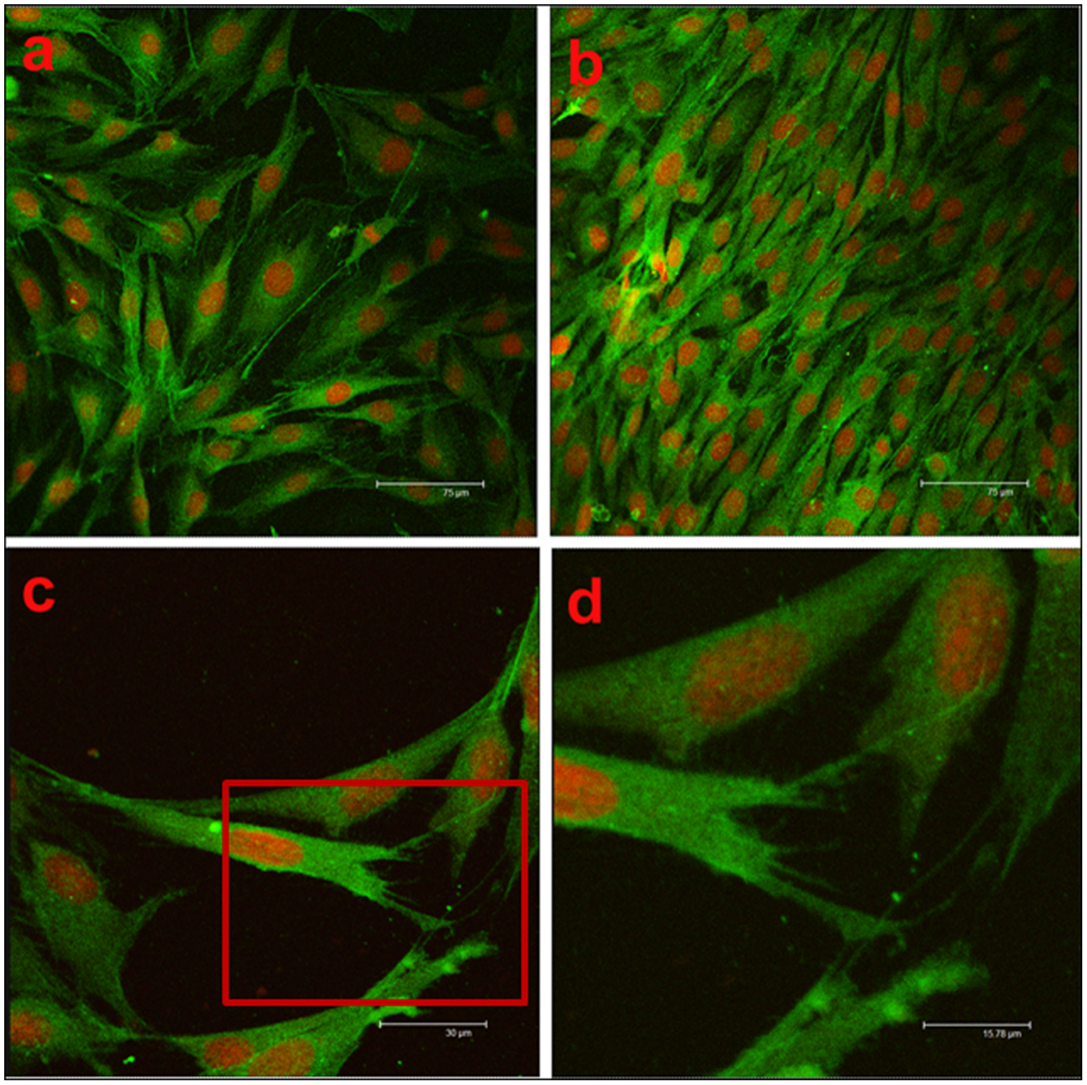
The morphology and distribution of cells cultured for 4 days on each scaffold. (a) PA66 scaffold (×200) (b) n-HA/PA66/GF scaffold (×200) (c) n-HA/PA66/GF scaffolds (×500) (d) higher magnification of cells corresponding to the rectangle region in (c). (×1000).

Figs. 4a–4d show the cytoskeletal organisation, attachment and spreading of MC3T3-E1 cells cultured on different substrates for 4 days. Cells (green) exhibited a long, spindle-shaped morphology, and the nuclei (red) were round or elliptical. The fluorescence staining of the cells was strong and well distributed. Cells connected with each other via fully stretched pseudopodia, which also cause the cells to attach tightly to the surface of the scaffold. The population of cells cultured with the n-HA/PA66/GF scaffold was larger than that of the PA66 scaffold culture, which demonstrated that the n-HA/PA66/GF scaffold had better biocompatibility and cell adhesion characteristics than PA66.

### 3. In vivo Evaluation

#### 3.1. General photography and image-based examination

**Figure 5:**
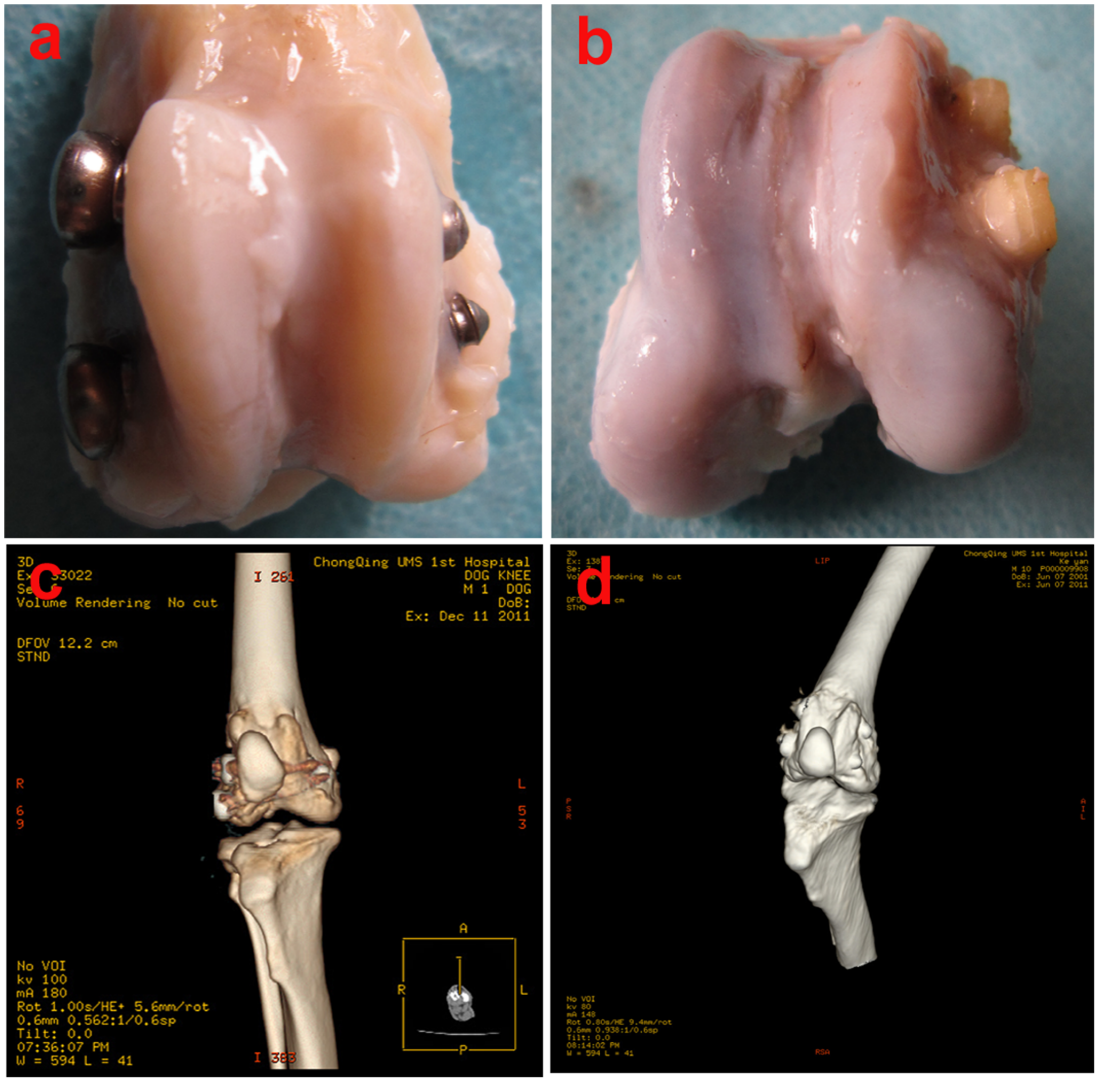
General photography and 3D-CT reconstruction images taken 12 weeks after operation. (a) General photography of metallic screws. (b) General photography of bioactive screws. (c) 3D-CT reconstruction image of metallic screws. (d) 3D-CT reconstruction image of bioactive screws.

Figs. 5a–5d shows that both types of screws could effectively fix the intercondylar fractures and that osteal healing occurred in both groups. In the experimental group, the bioactive screw fixed the fracture well, and the cortical bone became continuous. The primary fracture position could not be identified.

#### 3.2. Micro–CT examination

**Figure 6:**
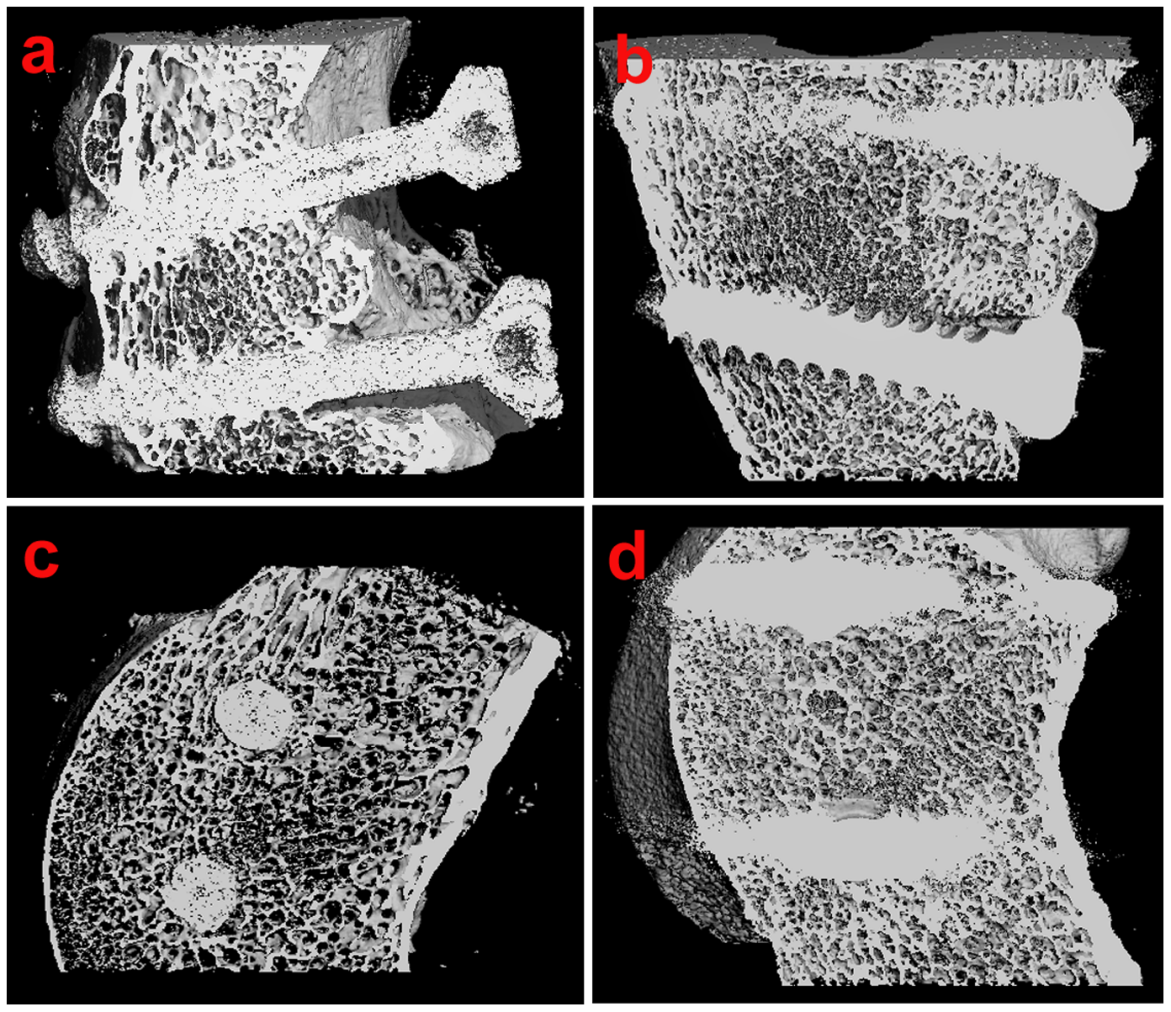
The micro-CT images of screws-bone interface taken at 12 weeks after operation. (a) Coronal micro-CT image of bioactive screw-bone interface. (b) Coronal micro-CT image of metallic screw-bone interface. (c) Sagittal micro-CT image of the bioactive screw-bone interface. (d) Sagittal micro-CT image of the metallic screw-bone interface.

Representative micro-CT images of the specimens are shown in Figs. 6a–6d. After 12 weeks, cortical bone became continuous with both types of screw fixation (Fig. 6a, 6b). The sagittal scanning images showed that the newly formed bone trabecula grew closely around the bioactive screws. The bioactive screws were tightly integrated with the host bone (Fig. 6c). However, in the control group, the image of bone trabecula around the metallic screws was vague because of metal artefacts (Fig. 6d).

#### 3.3. Histological analysis

**Figure 7:**
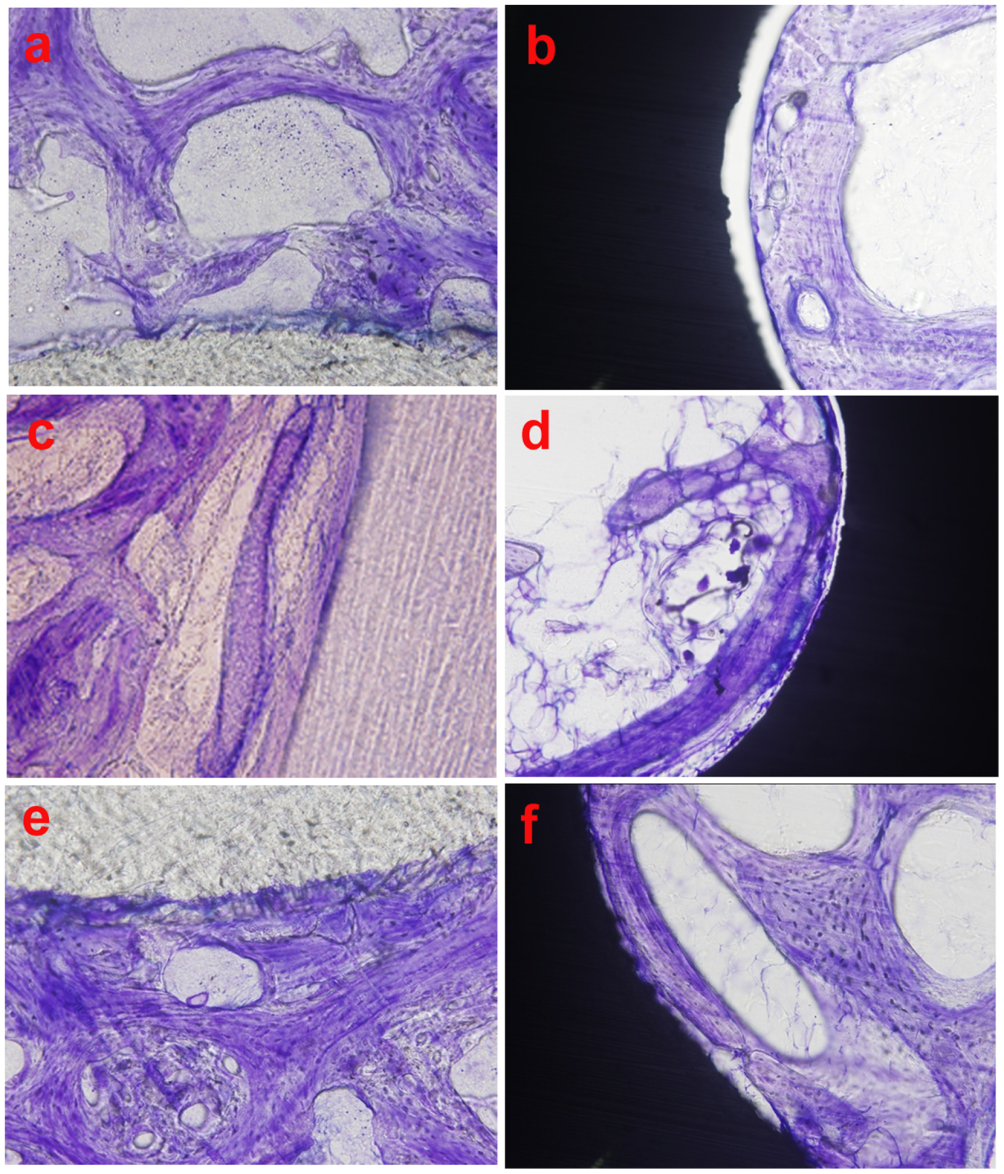
Toluidine blue (TB) staining of the screw-bone interface at different time points after operation (×200). (a) The bioactive screw-bone interface at 4 weeks after implantation. (b) The metallic screw-bone interface at 4 weeks after implantation. (c) The bioactive screw-bone interface at 8 weeks after implantation. (d) The metallic screw-bone interface at 8 weeks after implantation. (e) The bioactive screw-bone interface at 24 weeks after implantation. (f) The metallic screw-bone interface at 24 weeks after implantation.

Four weeks after implantation, newly formed bone collagen fibres and osteoids were observed around the bioactive screws, with no space between them (Fig. 7a). However, a transparent space appeared between the metallic screw and the host bone tissue (Fig. 7b). At 8 weeks after implantation, the newly formed bone around the bioactive screws had begun to calcify gradually into mature woven bone. The bioactive screws formed tight bonds with the surrounding bony tissue (Fig. 7c). In the control group, the space between the screw and the new bone decreased, however, the metallic screw still did not directly contact the surrounding bone tissue. The new bone surrounding the metallic screw was wrapped with a layer of fibrous tissue (Fig. 7d). At 24 weeks after implantation, a large amount of lamellar bone formed around the bioactive screw, and the bone was in direct contact with the bioactive screw (Fig. 7e). However, in the control group, the metallic screw still contacted the surrounding bone tissue less closely than the screw in the experimental group (Fig. 7f).

#### 3.4. Biomechanical assays

As shown in Table 2, the push-out strength of the screws increased with time after implantation. The maximum loads required for screw pullout from the host bone of the experimental group at 4, 8 and 12 weeks were similar to those of the control group. However, the maximum load of the experimental group at 24 weeks was significantly higher than that of the control group (*p*<0.05).

**Table 2:** The results of three-point bending tests in each period (n = 5, N, 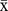 ± s).

Group	quantity	Observation time (h)			
		4w	8w	12w	24w
Experiment group	5	188.95±17.19^a^	268.94±37.27^a^	356.09±52.79^a^	528.28±63.71^b^
Control group	5	177.10±21.67	274.93±41.43	346.28±48.23	444.80±45.68

a:compared with control group, *P*>0.05;

b:compared with control group, *P*<0.05.

## Discussion

### 1. Composition and Mechanical Characteristics of the Screw

Hydroxyapatite [Ca_10_(PO4)_6_(OH)_2_], the major mineral component of human bone tissue, can promote bone in-growth because it has a Ca/P ratio within the range known to promote bone regeneration (1.50–1.67) [24]. HA is biocompatible and osteoinductive, and it is widely employed for hard tissue repair in orthopaedic surgery and dentistry [25], [26]. However, HA has poor mechanical stability, which limits its use for fixation in clinical practice. PA66 is an important engineering plastic with good biocompatibility and excellent mechanical properties. In addition, as a highly polar polymer, PA66 may form hydrogen bonds with nano-sized apatite [27]. Therefore, we combined HA with PA66 to obtain good mechanical strength. According to the results described by Wei [28], the high proportion of HA and uniform distribution of n-HA granules in the PA66 matrix give the scaffolding material good biocompatibility, high bioactivity and excellent mechanical strength. To meet the requirements of fracture fixation, we reinforced n-HA/PA66 with GF to improve the mechanical strength of the composite and used it to make screws.

According to our previous mechanical tests, the n-HA/PA66/GF screws have a bending strength of 200–300 MPa and an elastic modulus of 10–20 GPa. These values are similar to those of human cortical bone (120–210 MPa, 9–18 GPa) [8]. Therefore, the bioactive screws can fix the fractures firmly without stress-shielding. Because of their excellent biocompatibility, high mechanical strength and proper elastic modulus, the screws formed a firm bond with the new bone around them. Six months after implantation, the bioactive screws had integrated with the new bone without any sign of immunological rejection or fibrous tissue proliferation. Therefore, there was no need for a second operation to remove them.

### 2. Biocompatibility in vitro

The cell adhesion and spreading not only provide evidence of the cell–material interaction but also influence the subsequent performance of the implants. Sequential vital processes including proliferation, differentiation, maturation and ECM deposition occur after the cells initially adhere to the materials [29]. Therefore, we quantified the attachment and subsequent proliferation of MC3T3-E1 cells on the n-HA/PA66/GF scaffolds. The results showed that the n-HA/PA66/GF composite had no cytotoxicity and could support proper adhesion and proliferation of MC3T3-E1 cells, suggesting good in vitro cell adhesion and cytocompatibility.

F-actin is a major structural element of the cytoskeleton that is involved in a range of cell properties, including cell shape, mechanical stability and motility [30]. Increased F-actin expression is indicative of a better cell–substrate contact. Our results indicate that cells were induced to stretch on the substrate surface via pseudopodia formation and that focal adhesion proteins were recruited along the direction of the force exerted by the cytoskeleton, consistent with good cell adhesion. The increased adhesion and growth of MC3T3-E1 cells may be advantageous in promoting the growth of more new bone around implants and thus may increase implantation success rates.

### 3. The Screw Coating Layer

Improving the surface biocompatibility and bioactivity of bulk materials is a crucial topic in the biomedical field [31]. In this study, injection-moulded n-HA/PA66/GF screws were coated with n-HA. HA coating can increase bone-implant contact and the rate and amount of new bone formation around implant surfaces. Additionally, it can induce the rapid growth and ingrowth of the bone [32]. Therefore, in this study, we coated the screws with n-HA to achieve better bioactivity and osteogenesis. The results of mechanical testing showed that the bioactive screws possessed a similar holding force as metallic screws within three months of implantation. However, after three months, chemical bonds occurred between the n-HA coating and the host bone due to the excellent ability of the coating to promote bone formation and biocompatibility. Additionally, the HA coating existed as nanometre crystals. Nanometre-scale surface roughness has been shown to affect cell attachment and spreading [33]. Due to the “nanometre surface effect”, the area of the interface between the screw and the host bone increased greatly, which helped the bioactive screws form direct and stable bonds with the host bone. Therefore, our bioactive screws exhibited a higher holding force, surpassing that of metallic screws. In addition, more mature and complete Haversian systems formed around the bioactive screws earlier than around the metallic screws, demonstrating that the n-HA coating can promote maturity and increase the growth rate of newly formed bone. This study therefore introduces a successful approach for improving the clinical performance of screw implants.

In conclusion, our research group has produced a novel, non-degradable bioactive screw with excellent mechanical strength, biocompatibility and internal fixation properties. This bioactive screw promotes the healing of the host bone tissue and satisfies the fracture fixation requirements. Additionally, our results show that the interface between the bioactive screw and bone achieved good osteointegration and that the screws could provide rigid fixation without the need for a second operation.
